# Emerging Zoonotic Diseases: Should We Rethink the Animal–Human Interface?

**DOI:** 10.3389/fvets.2020.582743

**Published:** 2020-10-22

**Authors:** Ioannis Magouras, Victoria J. Brookes, Ferran Jori, Angela Martin, Dirk Udo Pfeiffer, Salome Dürr

**Affiliations:** ^1^Department of Infectious Diseases and Public Health, City University of Hong Kong, Kowloon, Hong Kong; ^2^School of Animal and Veterinary Sciences, Faculty of Science, Charles Sturt University, Wagga Wagga, NSW, Australia; ^3^Graham Centre for Agricultural Innovation (NSW Department of Primary Industries and Charles Sturt University), Wagga Wagga, NSW, Australia; ^4^UMR ASTRE (Animal, Santé, Territoires, Risques et Ecosystémes), CIRAD-INRAE, Université de Montpellier, Montpellier, France; ^5^Department of Philosophy, University of Basel, Basel, Switzerland; ^6^Vetsuisse Faculty, Veterinary Public Health Institute, University of Bern, Bern, Switzerland

**Keywords:** pandemic, zoonoses, emerging diseases, wildlife, bushmeat, one health

## Introduction

Humans have always been plagued by epidemics caused primarily by infectious diseases that originated from animals, especially wildlife (1). The establishment of sustained transmission from initial spillover events involves the interplay of complex mechanisms that are difficult to understand. However, there is consensus that direct or indirect contact of humans with animals and their body fluids (an “animal-human interface”) is essential for a successful cross-species transmission. Whilst humans have coexisted with domestic and wild animals for millennia, several anthropogenic factors have intensified the animal-human interface in recent decades, increasing our interactions with animals, and consequently, the risk of disease spillover. This increased intensity is largely driven by human population growth and efforts to alleviate the associated poverty, which include intensified farming and unsustainable exploitation of natural resources. Culinary traditions that include wildlife-meat consumption or traditional medicine also drive trade of wild animals, which can contribute to infectious disease emergence (2). In an increasingly globalized planet, a spillover event that results in an efficient and sustainable transmission between humans can spread very quickly. This has been well-demonstrated by the ongoing coronavirus disease (COVID-19) pandemic that resulted in an unprecedented global public health, social, and economic crisis. The current pandemic also illustrates that, despite our experiences with emerging zoonotic diseases (EZDs) such as Severe Acute Respiratory Syndrome (SARS), Ebola, and highly pathogenic H5N1 avian influenza, and subsequently improved national and global surveillance systems, humanity is not able to prevent new EZDs originating from animals. It is therefore crucial to re-evaluate potential sources of emerging pathogens at the animal-human interface and to examine whether we can minimize the risk for future pandemics at this point. We discuss important interfaces that drive zoonotic disease emergence and spread, and then discuss the feasibility of reducing the risks of EZDs at these interfaces.

## Wet Markets and Other Live Animal Markets

The definition of a “wet market” can vary with context. Here, we refer to fresh-food markets in which live animals are sold, most commonly for food or medicine, and are slaughtered at the market. This type of market is common throughout Asia, where live animals such as fish, crustaceans, poultry (live bird market sections), various mammals, and other fresh products such as vegetables, are sold. Despite the rapid expansion of supermarkets in Asia, studies have shown that up to 77% of consumers choose wet markets as their primary source of fresh food because they prefer fresh meat (3, 4).

Wet markets have been stigmatized in recent years due to their association with potential infectious disease emergence, such as avian influenza transmission in live bird markets (5, 6). Some wet markets also sell wild animals (wildlife markets), such as reptiles, porcupines, and other species. The SARS virus outbreak (2002–2003) that killed 774 people likely originated from masked palm civets (*Paguma larvata*) sold in wildlife markets in Guangdong Province, China (7). The ongoing COVID-19 pandemic is thought to have originated at the Huanan seafood wholesale market in Wuhan (China). This seafood market also sold live wild animals, such as several species of birds, reptiles, and small mammals, suggesting a possible zoonotic transmission from wildlife to humans (8). Eating wild animals is a symbol of wealth, and their meat is perceived to be more natural and nutritious than meat from farmed animals, and is also an ingredient in traditional medicines (9). Wet markets are often characterized by poor hygiene (10, 11) and the presence of live animals kept in crowded conditions. Together with the difficulty of hygienically selling food in such environments, another risk factor for EZDs is the largely undescribed virus diversity that can been found in some wildlife orders, such as bats, rodents and primates (12). Wild animals that have been removed from their natural habitat, and are housed in conditions that do not promote their welfare, will suffer from severe stress, potentially causing immunosuppression and shedding of pathogens that they may be carrying (13, 14). Despite the warning of the SARS outbreak, COVID-19 emergence has demonstrated that the consumption of freshly slaughtered meat and wildlife is an entrenched activity, and therefore, the sale of live animals, including wildlife, in markets is resistant to change.

## Wildlife Hunting and Consumption

Hunting and gathering only started to be replaced by livestock breeding and agriculture about 10,000 years ago (15). In some regions of the world—mainly in the tropics where livestock is poorly developed—wildlife hunting and consumption is still commonly practiced, with such meat known as “bushmeat,” particularly in Africa (16). In these contexts, wildlife represent a major source of protein and/or income through the sale of meat, large-game tourism (17–19) and trading products for traditional medicine (20), and are also valued for traditional hunting and ceremonial events (21–23). In this context, any activity manipulating wildlife species provides an animal-human interface facilitating a potential pathogen spillover (24). Hunters (mainly men), as well as any person handling dead animals during trade and cooking (mainly women), are exposed to potential pathogens present in animal carcasses and their body fluids. The human immune-deficiency virus (HIV) originated from non-human primates and it is suggested that contacts with hunted primates were responsible for the spillover of this virus to humans (25, 26). Bushmeat consumption has also been implicated in the emergence of Ebola virus disease, resulting in several outbreaks in Central Africa over the last five decades, as well as the large epidemic in West Africa in 2013–2016 in which over 11,300 people died (27, 28). Fruit bats were identified as a reservoir species and spillover to humans happened directly or indirectly *via* an intermediate wildlife species (29, 30). For example, in the West African outbreak, a single spillover event from a fruit bat to a human was suspected to have resulted in sustained human transmission without further animal involvement (28). In contrast, in some of the Ebola virus outbreaks in Central Africa, chimpanzee, or gorilla carcasses were identified as sources of human infection (27), highlighting the important role of species that are closely related to humans for zoonotic spillover.

## Intensive Wildlife Farming

Several species of mammals—for example, deer (31), rodents (32), civets (33), and fur mammals—are bred under a wide range of production systems worldwide, and provide income and protein. The legal and technical framework for these production systems is often poor (33, 34) and published information on the biology, production and health of these non-conventional captive species is scarce, particularly in low-income countries (35). Consequently, health-monitoring programs in wildlife farms are seldom implemented, despite intensive farming conditions and low genetic diversity (34, 36). These factors expose farmed wildlife species to stress and immunosuppression (13), and predispose captive wild animal populations to disease emergence. This is illustrated by the circulation of avian influenza strains in ostrich (*Struthio camelus*) farms in South Africa (37), the occurrence of repeated rabies outbreaks in ranched kudu *(Tragelaphus strepsiceros)* populations in Namibia (38), and the recent detection of SARS-CoV-2 circulation in mink (*Neovison vison*) farms in the Netherlands (39).

## Domestic Animals—Livestock and Pets

Although there are relatively few domesticated species, livestock and companion animals have interfaces with both wildlife and people, and therefore have an important role in the complex pathways leading to EZDs.

Intensive livestock farming is increasing worldwide, encouraged by market demands including urbanization and expanding global populations which have changed the way in which food is produced and supplied (40). Concurrent anthropogenic factors, such as changes in land-use, provide new wildlife-domestic species interfaces by creating shared ecologies, with opportunity for spillover and amplification of new EZDs (41). Nipah virus emergence in Malaysia in 1998 is one such example (42). Dual–agriculture of intensive pig farming with mango plantations created a bat-pig interface that allowed spillover of Nipah virus from bats feeding on the fruit trees to pigs housed below. Repeated spillover events from bats resulted in prolonged circulation of the virus in pigs, increasing the opportunity for spillover to people (43). This illustrates that large, dynamic populations of a single livestock species can increase the risk of EZDs in people by enabling persistence of a potential pathogen at the livestock-human interface. Mixing of domestic species can also give rise to EZDs; for example, avian influenza viruses circulate and re-combine in domestic poultry in live-bird markets (44). Examples in which companion animal species have provided an interface for EZDs between wildlife and people include Hendra virus (45) and *Chlamydia psittaci* (46). These examples illustrate multiple epidemiologic scenarios involving individual, mixed, or large dynamic domestic animal populations that provide an intermediate interface between wildlife and humans.

## Discussion

**Figure 1:**
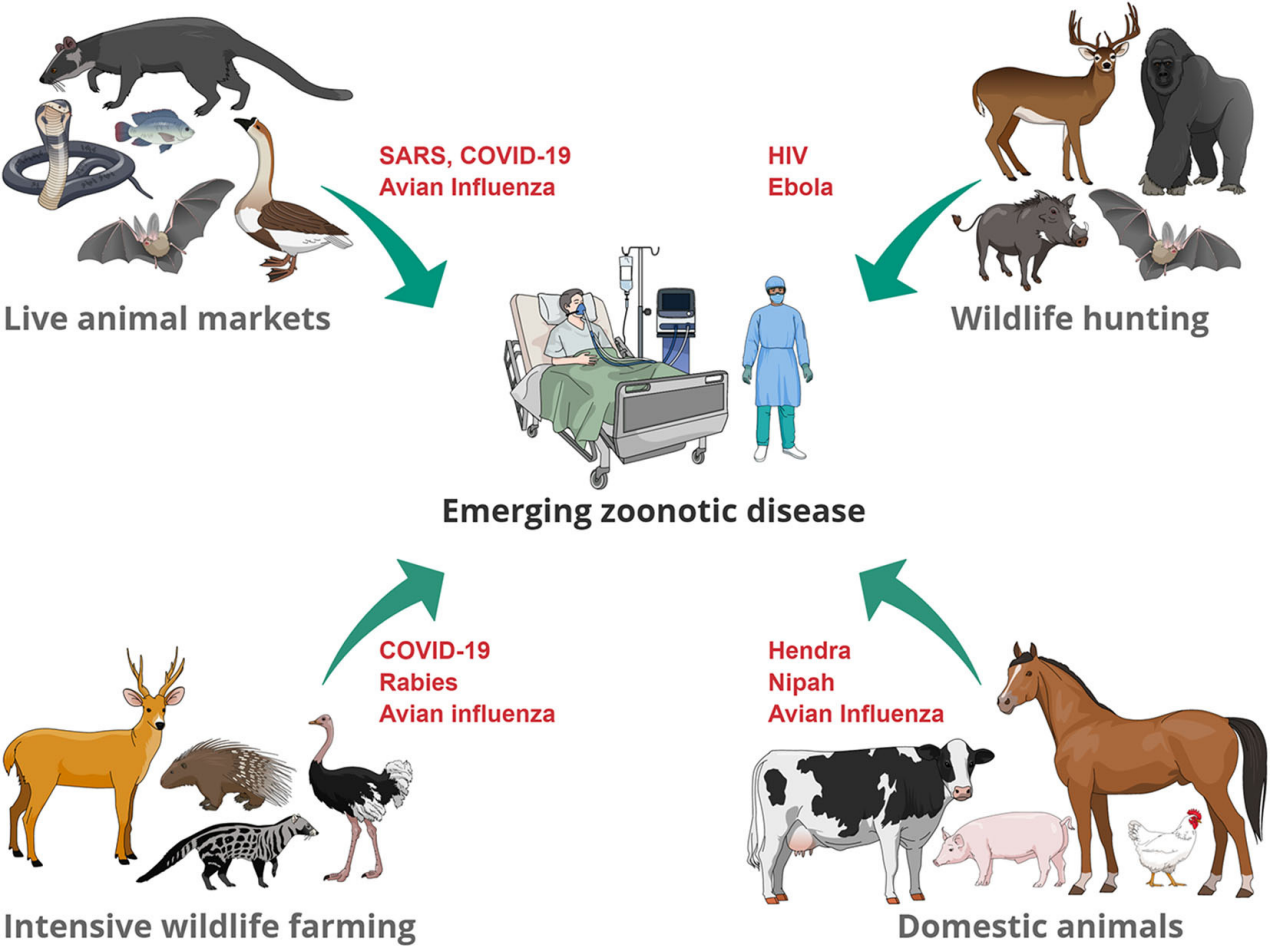
Examples of zoonotic diseases that have (re-) emerged at the animal-human interface. Transmission pathways include direct contact through handling of living animals (wildlife trade, domestic animals), preparation of slaughtered animals for consumption of meat or for traditional medicine uses.

Our presentation of the different interfaces and potential sources of EZD (Figure 1), demonstrates a recurring theme of intensified anthropogenic factors driven by cultural and socioeconomic interests. The challenges for many of these interfaces include achieving a balance between sustainably managing resources required for human population growth, safeguarding species conservation and biodiversity, securing animal and human health, and respecting animal welfare, when large numbers of species are kept in confined spaces (for example, farms and markets). Such use of animals also gives rise to ethical questions related to animal husbandry. Animals' fundamental interests should not be sacrificed if it were not for weightier human interests. This means that the use of animals is, in some contexts, morally permissible (for example, when there is no healthy plant-based alternative to meat), while in other contexts, it is morally problematic (for example, when wild animals are traded and consumed as a symbol of wealth). Whilst it is unrealistic to expect immediate changes in the way humans exploit animals without addressing underlying drivers of this behavior, more consideration should be given to the living conditions of animals in intensive livestock/wildlife farms and in live-animal markets. Less crowded living conditions and respect for biological and behavioral needs of species (such as foraging and occupational opportunities) will not only improve the animals' wellbeing, but also result in lower stress and therefore lower risk of spillover.

Popular reactions to EZD emergence often target the immediate source, rather than underlying drivers. For example, some have suggested shutting down wildlife markets (47). However, as the drivers of wild-animal meat consumption will persist even after a global health crisis, this is likely to shift the interface elsewhere, out of sight of the regulators (48–50). In our opinion, such bans could lead to the emergence of further illegal, unregulated wildlife markets and increased poaching, which would make it impossible to monitor market dynamics, develop surveillance systems, and implement risk mitigation measures. In addition, the interconnectedness of the different interfaces discussed here is illustrated when wildlife hunting is replaced by livestock farming. Some farming practices result in deforestation of large areas (51), and this in turn provides a livestock-wildlife interface and therefore, the potential risk for pathogen spillover from wildlife to livestock. The interconnectedness and complexity of these ecologies demonstrates the need for that holistic approaches according to the One Health and Planetary Health concepts. Both concepts follow the principle that human, animal, and environmental health cannot be separated, and therefore, to solve health problems, all three health fields and the sustainable use of natural resources have to be considered (52).

The focus has been on early detection and rapid response to EZDs in our efforts to control their impacts. Epidemiologists have many tools that can be integrated—for example, horizon scanning, prioritization, and disease modeling—to provide a greater awareness of the EZDs, as well as provide insights for their control (53). Whilst many improvements using integrated tools can still be made across systems for disease preparedness (54), we now also call for actions to reduce the rate of EZDs at the human–animal interfaces. Such actions could include improving hygiene, animal welfare, disease surveillance and safeguarding species conservation through comprehensive and culturally tailored regulations. This will require, in many circumstances, a greater understanding of the sociocultural drivers. For example, the application of social and ethnographic sciences could provide insights about the sociocultural context of wildlife exploitation and trade, and identify potential solutions to promote healthier bushmeat consumption and trade, particularly in tropical forest regions in which livestock farming is poorly developed (35).

In addition, wildlife production systems should be supervised and monitored by international bodies in a comparable way as international certification agencies already control forestry exploitation activities to ensure sustainable wood exploitation (32). Similarly, we need regulation of wildlife farms in the same way that mainstream agriculture is regulated to control welfare and biosecurity conditions. Although wildlife farms represent a minor contribution to national economies, they can have important implications in terms of public health (and we have now seen how that affects economies). Also, alternative protein sources such as aquaculture should be explored; the large diversity of farmed species in aquaculture provides a wide range of opportunities for many countries, while the risk of zoonotic disease emergence is negligible when compared with terrestrial species.

Due to the anthropogenic nature of drivers of EZDs (increased human population, globalization, climate change) changes require government-level strategies that are integrated globally, as well as raising awareness through targeted education of stakeholders including consumers and farmers to improve pathogen surveillance, animal welfare, and reduce environmental impacts of livestock and wildlife farming. With massive human population growth, globalization of trade and travel, and unsustainable use of natural resources, humanity is in a critical phase in which we head toward irreversible global crises. The more we focus on our short-term anthropocentric model of development, the more our coexistence becomes disconnected from nature. This has been proven to have serious and devastating consequences for humankind, such as the impact of EZDs, and for the planet (52). As demonstrated here, the challenges associated with risk mitigation and control of EZDs are tightly interlinked with global sustainability. We therefore appeal for more sustainable animal harvesting and production practices, with a stronger focus on health, and not solely productivity. This will not only reduced the risk for EZDs, but also improve environmental balance and animal welfare.

## Author Contributions

IM and SD conceptualized the manuscript. IM wrote the introduction, the part about wet markets and other live animal markets and designed the figure. VB wrote the part about domestic animals-livestock and pets. FJ wrote the part about intensive wildlife farming. AM provided input on animal ethics. DP provided input on all topics and the discussion. SD wrote the part about wildlife hunting and consumption and the main body of the discussion. All the authors contributed in the discussion and editing of the manuscript. All the authors have read and approved the manuscript.

## Conflict of Interest

The authors declare that the research was conducted in the absence of any commercial or financial relationships that could be construed as a potential conflict of interest.
